# Polydeoxyribonucleotide Exerts Protective Effect Against CCl_4_-Induced Acute Liver Injury through Inactivation of NF-κB/MAPK Signaling Pathway in Mice

**DOI:** 10.3390/ijms21217894

**Published:** 2020-10-24

**Authors:** Il-Gyu Ko, Jun-Jang Jin, Lakkyong Hwang, Sang-Hoon Kim, Chang-Ju Kim, Jin Hee Han, Seunghwan Lee, Ha Il Kim, Hyun Phil Shin, Jung Won Jeon

**Affiliations:** 1 Department of Physiology, College of Medicine, Kyung Hee University, Seoul 02447, Korea; rhdlfrb@naver.com (I.-G.K.); threej09@hanmail.net (J.-J.J.); lhwangphd@gmail.com (L.H.); kkmeksh@gmail.com (S.-H.K.); changju@khu.ac.kr (C.-J.K.); 2 Department of Anesthesiology and Pain Medicine, College of Medicine, Kyung Hee University, Seoul 02447, Korea; esthesi@khu.ac.kr; 3 Department of Surgery, Kyung Hee University Hospital at Gangdong, College of Medicine, Kyung Hee University, Seoul 05278, Korea; histones@hanmail.net; 4 Department of Internal Medicine, Kyung Hee University Hospital at Gangdong, College of Medicine, Kyung Hee University, Seoul 05278, Korea; mondosewan@gmail.com (H.I.K.); drshp@khu.ac.kr (H.P.S.)

**Keywords:** acute liver injury, polydeoxyribonucleotide, nuclear factor-kappa B, mitogen-activated protein kinase, inflammation, apoptosis

## Abstract

Acute liver injury (ALI) causes life-threatening clinical problem, and its underlying etiology includes inflammation and apoptosis. An adenosine A_2A_ receptor agonist, polydeoxyribonucleotide (PDRN), exhibits anti-inflammatory and anti-apoptotic effects by inhibiting the secretion of pro-inflammatory cytokines. In the current study, the protective effect of PDRN against carbon tetrachloride (CCl_4_)-induced ALI was investigated using mice. For the induction of ALI, mice received intraperitoneal injection of CCl_4_ twice over seven days. Mice from the PDRN-treated groups received an intraperitoneal injection of 200 μL saline containing PDRN (8 mg/kg), once a day for seven days, starting on day 1 after the first CCl_4_ injection. In order to confirm that the action of PDRN occurs through the adenosine A_2A_ receptor, 8 mg/kg 3,7-dimethyl-1-propargylxanthine (DMPX), an adenosine A_2A_ receptor antagonist, was treated with PDRN. Administration of CCl_4_ impaired liver tissue and increased the liver index and histopathologic score. The expression of pro-inflammatory cytokines was increased, and apoptosis was induced by the administration of CCl_4_. Administration of CCl_4_ activated nuclear factor-kappa B (NF-κB) and facilitated phosphorylation of signaling factors in mitogen-activated protein kinase (MAPK). In contrast, PDRN treatment suppressed the secretion of pro-inflammatory cytokines and inhibited apoptosis. PDRN treatment inactivated NF-κB and suppressed phosphorylation of signaling factors in MAPK. As a result, liver index and histopathologic score were reduced by PDRN treatment. When PDRN was treated with DMPX, the anti-inflammatory and anti-apoptotic effect of PDRN disappeared. Therefore, PDRN can be used as an effective therapeutic agent for acute liver damage.

## 1. Introduction

The liver is responsible for many physiological functions, including metabolism. However, exogenous substances such as alcohol, drugs, chemicals, and infections can cause liver toxicity and ultimately lead to liver damage [1,2]. Acute liver injury (ALI) is a sudden liver damage that occurs within a short period of time. ALI causes life-threatening clinical problems such as blood clotting disorders and high mortality [3]. Acute liver failure refers to severe ALI with hepatic encephalopathy and coagulation dysfunction [4].

The causes of ALI include complex interactions of oxidative stress, apoptosis, and inflammation [5]. The mechanism of cell death is to keep cells functioning normally by removing cells that are harmful or severely damaged [6]. However, excessive hepatocyte apoptosis is likely to contribute to ALI by causing liver dysfunction [7]. Natural killer cells, T cells, and macrophages are mobilized during ALI, and liver-resident macrophages act as a factor contributing to liver damage by mass production of cytokines in response to inflammatory stimuli [8]. These inflammatory cells secrete pro-inflammatory cytokines such as tumor necrosis factor-α (TNF-α), interleukin (IL)-1β, and IL-6, and these pro-inflammatory cytokines play a role in promoting ALI progression. During ALI progression, various damaging factors contribute to the inflammatory response and promote liver cell apoptosis, thereby increasing liver tissue damage [9,10].

The nuclear factor-κB (NF-κB) activation and active forms of the mitogen-activated protein kinase (MAPK) pathway regulate the expression of pro-inflammatory cytokines [11,12]. Expression of TNF-α, IL-1β and IL-6 is induced by activation of NF-κB [13]. Extracellular signal-regulated kinase (ERK), c-Jun N-terminal kinase (JNK), and p38 constitute MAPK, which is regulated by NF-κB through a variety of pathways [14,15]. Inflammation and apoptosis are also regulated by MAPK [14,16]. Due to the action of NF-κB and MAPK, they have been proposed as targets of the therapeutic pathway of ALI [16,17].

G protein-coupled receptors expressed in immune cells, A_1_, A_2A_, A_2B_, and A_3_, mediate various actions of adenosine [12]. Of these, the adenosine A_2A_ receptor is an important component of an endogenous “immunosuppressive loop” and is found in most cells involved in wound healing [18,19]. Adenosine A_2A_ receptor agonist polydeoxyribonucleotide (PDRN) was extracted from salmon sperm, and PDRN has an anti-inflammatory effect by inhibiting the production of pro-inflammatory cytokines. Stimulation of A_2A_ by PDRN has been reported to reduce the secretion of pro-inflammatory cytokines and inhibit apoptotic cell death in various disease states [12,19,20,21]. PDRN promotes damage healing and inhibits inflammation, but the mechanism of PDRN treatment for liver damage has not been clearly elucidated. Therefore, in this study, we evaluated the effect of PDRN on carbon tetrachloride (CCl_4_)-induced ALI focusing on the NF-κB and MAPK signaling pathway.

The liver index and the level of alanine aminotransferase (ALT) and aspartate aminotransferase (AST) were measured. Hematoxylin and eosin (H&E) staining was performed to determine liver histopathological score. Concentration of TNF-α, IL-1β, IL-6, and cyclic adenosine-3′,5′-monophosphate (cAMP) was detected using enzyme-linked immunoassay (ELISA). Western blot analysis was conducted for the determination of cytochrome P450 2E1 (CYP2E1), uncoupling protein 2 (UCP2), NF-κB, NF-κB inhibitor-α (IκB-α), ERK, JNK, p38, cAMP response element-binding protein (CREB), protein kinases A (PKA), Bcl-2-associated X protein (Bax), B-cell lymphoma-2 (Bcl-2), cleaved caspase-3-positive cells, and cleaved caspase-9-positive cells.

## 2. Results

### 2.1. Level of AST and ALT, Expression of CYP2E1 and UPC2

**Figure 1:**
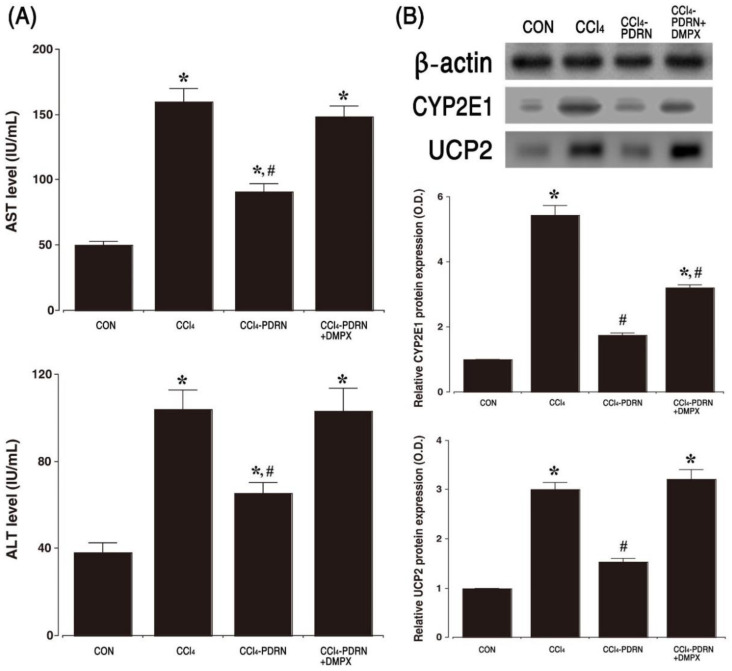
Level of aspartate aminotransferase (AST) and alanine aminotransferase (ALT), and expression of cytochrome P450 2E1 (CYP2E1) and uncoupling protein 2 (UCP2). (**A**) Concentration of AST and ALT in serum. (**B**) Representative expression of CYP2E1 and UCP2 in liver tissue. CON, control group; CCl_4_, CCl_4_-injection group; CCl_4_-PDRN, CCl_4_-injection and polydeoxyribonucleotide (PDRN)-treated group; CCl_4_-PDRN+DMPX, CCl_4_-injection and PDRN with 3,7-dimethyl-1-propargylxanthine (DMPX)-treated group. * indicates *p* < 0.05 when compared with the control group. ^#^ indicates *p* < 0.05 when compared with the CCl_4_-injection group.

The concentration of AST and ALT in CCl_4_-induced ALI mice is presented in Figure 1A. The concentration of serum AST and ALT was enhanced by intraperitoneal administration of CCl_4_ (*p*< 0.05). In contrast, PDRN treatment reduced serum AST and ALT concentration (*p* < 0.05). The relative expression of CYP2E1 and UCP2 was measured by Western blot analysis (Figure 1B). Intraperitoneal administration of CCl_4_ increased the expression of CYP2E1 and UCP2 in liver tissue compared to the control group (*p* < 0.05). However, PDRN treatment decreased the expression of CYP2E1 and UCP2 in liver tissue (*p* < 0.05).

To evaluate the adenosine A_2A_ receptor involvement of PDRN efficacy, the inhibitory effect of PDRN on AST, ALT, CYP2E1, and UCP2 disappeared after co-treatment of DMPX with PDRN.

### 2.2. Liver Morphology and Histopathological Score

**Figure 2:**
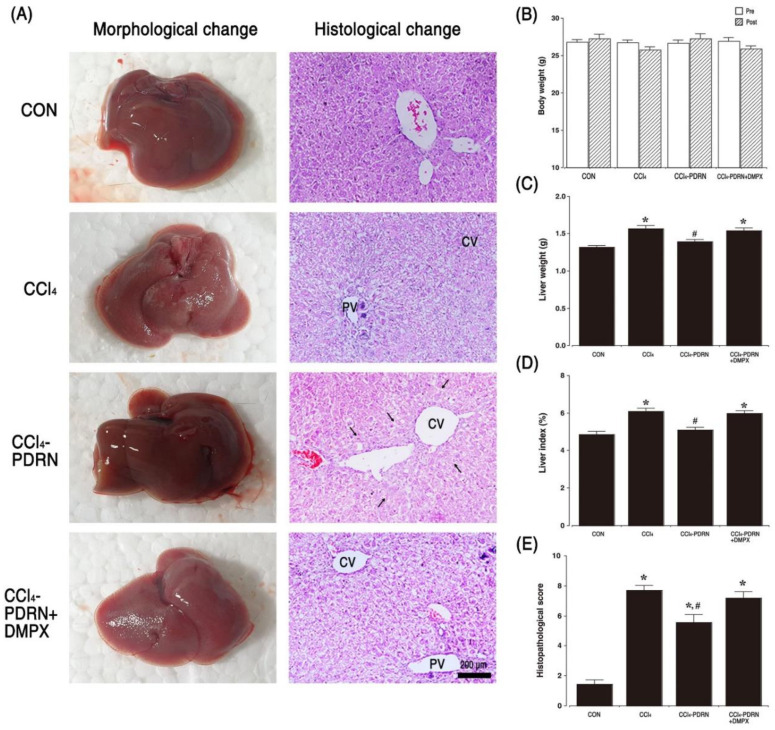
Change in morphology, histology, and liver index. (**A**) Morphological and histological change in the liver. (**B**) Body weight change during the experiment. (**C**) Liver weight in each group. (**D**) Liver index in each group. (**E**) Histopathological score in each group. (CV) Central vein, (PV) portal vein. Block arrows indicate recovery from hepatocyte necrosis. □: Measurement before experiment, ▨: Measurement at the end of the experiment. CON, control group; CCl_4_, CCl_4_-injection group; CCl_4_-PDRN, CCl_4_-injection and polydeoxyribonucleotide (PDRN)-treated group; CCl_4_-PDRN+DMPX, CCl_4_-injection and PDRN with 3,7-dimethyl-1-propargylxanthine (DMPX)-treated group. * indicates *p* < 0.05 when compared with the control group. ^#^ indicates *p* < 0.05 when compared with the CCl_4_-injection group.

Figure 2 shows the morphological and histological properties of CCl_4_-induced ALI. Intraperitoneal administration of CCl_4_ altered gross morphology of the liver, such as scattered white granules, gray color, brittle texture, and liver enlargement. The liver weight and liver index were increased due to these changes (*p* < 0.05). Histological analysis showed that exposure to CCl_4_ caused damage in the liver parenchyma, resulting in centrilobular or periportal necrosis, apparent hepatic edema, infiltration of inflammatory cells, destruction of cord-like arrangement of hepatocyte, and acute hepatocyte degeneration.

These changes increased the liver histopathological scores (*p* < 0.05). In contrast, PDRN treatment alleviated these changes and reduced liver weight, liver index, and liver histopathological score (*p* < 0.05). To evaluate the adenosine A_2A_ receptor involvement of PDRN efficacy, the inhibitory effect of PDRN on liver weight, liver index, and liver histopathological score disappeared after co-treatment of DMPX with PDRN.

### 2.3. Level of Pro-Inflammatory Cytokines

**Figure 3:**
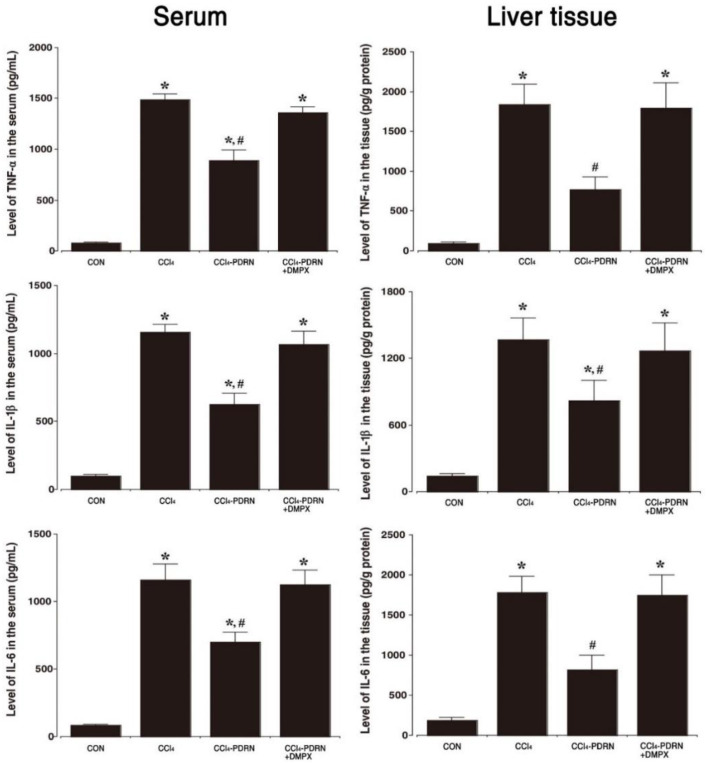
Level of pro-inflammatory cytokines. Left panel: Concentration of tumor necrosis factor (TNF)-α, interleukin (IL)-1β, and IL-6 in serum. Right panel: Expression of TNF-α, IL-1β, and IL-6 in liver tissue. CON, control group; CCl_4_, CCl_4_-injection group; CCl_4_-PDRN, CCl_4_-injection and polydeoxyribonucleotide (PDRN)-treated group; CCl_4_-PDRN+DMPX, CCl_4_-injection and PDRN with 3,7-dimethyl-1-propargylxanthine (DMPX)-treated group. * indicates *p* < 0.05 when compared with the control group. ^#^ indicates *p* < 0.05 when compared with the CCl_4_-injection group.

The concentration of TNF-α, IL-1β, and IL-6 in serum and liver tissue is shown in Figure 3. In the CCl_4_-induced ALI, the concentration of TNF-α, IL-1β, and IL-6 in serum and liver tissue was increased (*p* < 0.05). In contrast, treatment with PDRN reduced the concentration of TNF-α, IL-1β and IL-6 in serum and liver tissue (*p* < 0.05). To evaluate the adenosine A_2A_ receptor involvement of PDRN efficacy, the inhibitory effect of PDRN on the production of pro-inflammation cytokines disappeared after co-treatment of DMPX with PDRN.

### 2.4. NF-κB Activation and MAPK Signaling Pathway

**Figure 4:**
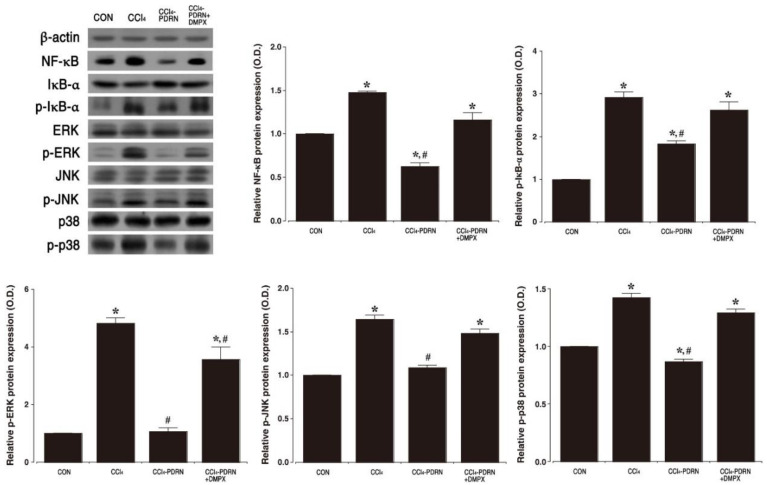
Expression of nuclear factor (NF)-κB and cascade factors of mitogen-activated protein kinase (MAPK). Upper left panel: Representative expression of NF-κB, NF-κB inhibitor (IκB)-α, extracellular signal-regulated kinase (ERK), c-Jun N-terminal kinase (JNK), p-38. Upper right panel: Relative expression of NF-κB and relative ratio of phosphorylated IκB-α (p-IκB-α) to IκB-α. Lower panel: Relative ratio of phosphorylated ERK (p-ERK) to ERK, phosphorylated JNK (p-JNK) to JNK and phosphorylated p-38 (p-p-38) to p-38. CON, control group; CCl_4_, CCl_4_-injection group; CCl_4_-PDRN, CCl_4_-injection and polydeoxyribonucleotide (PDRN)-treated group; CCl_4_-PDRN+DMPX, CCl_4_-injection and PDRN with 3,7-dimethyl-1-propargylxanthine (DMPX)-treated group. * indicates *p* < 0.05 when compared with the control group. ^#^ indicates *p* < 0.05 when compared with the CCl_4_-injection group.

The effect of PDRN on activation of NF-κB and phosphorylation of MAPK signaling pathway was investigated by Western blot (Figure 4). Intraperitoneal administration of CCl_4_ increased phosphorylation of IκB-α and enhanced NF-κB expression in liver tissue (*p* < 0.05), indicating activation of NF-κB. In contrast, PDRN treatment suppressed phosphorylation of IκB-α and decreased NF-κB expression in liver tissue (*p* < 0.05), indicating inactivation of NF-κB. Intraperitoneal administration of CCl_4_ increased the phosphorylation of ERK, JNK and p38 of the MAPK signaling pathway in liver tissue (*p* < 0.05). In contrast, PDRN treatment mitigated phosphorylation of ERK, JNK and p38 in liver tissue (*p* < 0.05). To evaluate the adenosine A_2A_ receptor involvement of PDRN efficacy, the inhibitory effect of PDRN on the phosphorylation of IκB-α, ERK, JNK and p38 disappeared after co-treatment of DMPX with PDRN.

### 2.5. cAMP Concentration, P-CREB vs. CREB Ratio, and P-PKA vs. PKA Ratio

**Figure 5:**
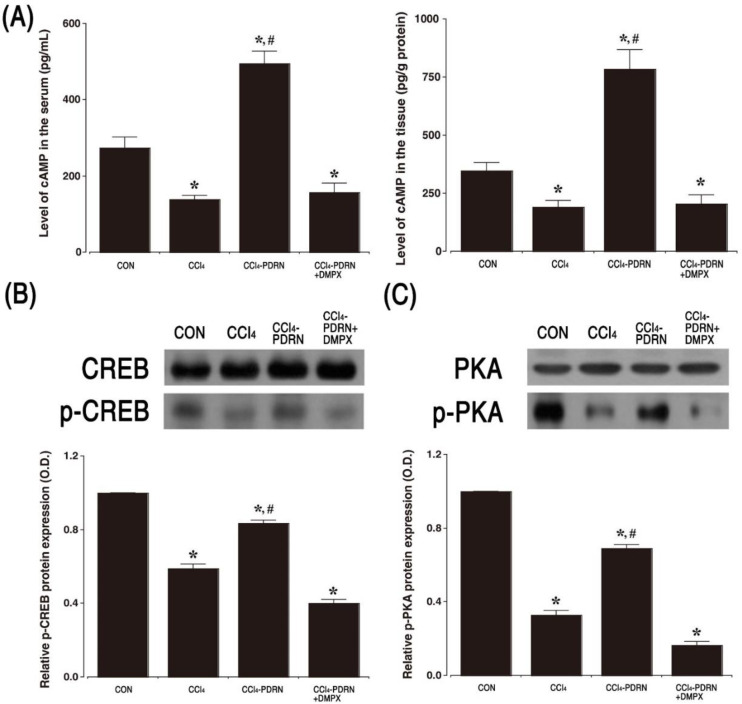
Expression of cyclic adenosine-3′,5′-monophosphate (cAMP), cAMP response element-binding protein (CREB) and protein kinase A (PKA). (**A**) Concentration of cAMP in serum and liver tissue. (**B**) Relative ratio of phosphorylated CREP (p-CREB) to CREB in liver tissue. (**C**) Relative ratio of phosphorylated PKA (p-PKA) to PKA in liver tissue. CON, control group; CCl_4_, CCl_4_-injection group; CCl_4_-PDRN, CCl_4_-injection and polydeoxyribonucleotide (PDRN)-treated group; CCl_4_-PDRN+DMPX, CCl_4_-injection and PDRN with 3,7-dimethyl-1-propargylxanthine (DMPX)-treated group. * indicates *p* < 0.05 when compared with the control group. ^#^ indicates *p* < 0.05 when compared with the CCl_4_-injection group.

The cAMP concentration in serum and liver tissue is shown in Figure 5A. Intraperitoneal administration of CCl_4_ decreased cAMP concentration in serum and liver tissue (*p* < 0.05). In contrast, PDRN treatment increased the concentration of cAMP in serum and liver tissue (*p* < 0.05). The relative expression of p-CREB vs. CREP and p-PKA vs. PKA was measured by Western blot analysis (Figure 5B,C). Intraperitoneal administration of CCl_4_ reduced the ratio of p-CREB vs. CREB and p-PKA vs. PKA in liver tissue compared to the control group (*p* < 0.05). In contrast, PDRN treatment improved the ratio of p-CREB vs. CREB and p-PKA vs. PKA in liver tissue (*p* < 0.05). To evaluate the adenosine A_2A_ receptor involvement of PDRN efficacy, the improving effect of PDRN on cAMP concentration and phosphorylation of CREB and PKA disappeared after co-treatment of DMPX with PDRN.

### 2.6. Cleaved Caspase-3 and Cleaved Caspase-9 Expression, and Bax vs. Bcl-2 Ratio

**Figure 6:**
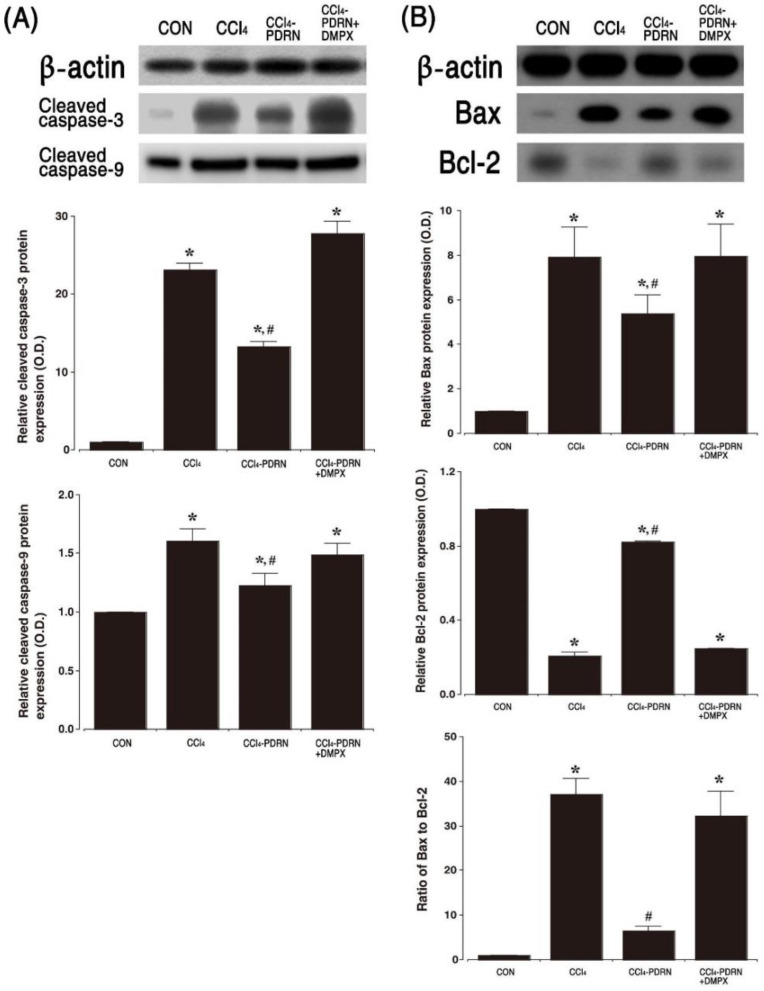
Cell death by apoptosis in liver tissue. (**A**) Representative expression of cleaved caspase-3 and cleaved caspase-9 in liver tissue. (**B**) Relative ratio of Bcl-2-associated X protein (Bax) to B-cell lymphoma-2 (Bcl-2) in liver tissue. CON, control group; CCl_4_, CCl_4_-injection group; CCl_4_-PDRN, CCl_4_-injection and polydeoxyribonucleotide (PDRN)-treated group; CCl_4_-PDRN+DMPX, CCl_4_-injection and PDRN with 3,7-dimethyl-1-propargylxanthine (DMPX)-treated group. * indicates *p* < 0.05 when compared with the control group. ^#^ indicates *p* < 0.05 when compared with the CCl_4_-injection group.

Western blot analysis for Bax, Bcl-2, cleaved caspase-3 and cleaved caspase-9 expression is presented in Figure 6. Intraperitoneal administration of CCl_4_ increased cleaved caspase-3 and cleaved caspase-9 expression (*p* < 0.05). The Bax vs. Bcl-2 ratio was also improved by intraperitoneal administration of CCl_4_ (*p* < 0.05). In contrast, PDRN treatment inhibited cleaved caspase-3 and cleaved caspase-9 expression, and the Bax vs. Bcl-2 ratio (*p* < 0.05). To evaluate the adenosine A_2A_ receptor involvement of PDRN efficacy, the inhibitory effect of PDRN on apoptosis disappeared after co-treatment of DMPX with PDRN.

## 3. Discussion

CCl_4_ has been used extensively to create animal models of severe liver damage by the generation of oxidative stress and activation of immune cells [11]. Therefore, in an experimental animal model, CCl_4_ was chosen as a drug that causes liver damage. Serum activity such as ALT and AST is a very sensitive marker used in the diagnosis of liver disease [1]. In particular, an increased level of serum AST and ALT is a major indicator of CCl_4_-induced liver toxicity with cell membrane damage and loss of hepatocyte functional integrity [10].

CCl_4_ is mainly metabolized to highly reactive trichloromethyl radicals by CYP2E1. These reactive free radicals induce liver damage by triggering a chain of cellular events [22]. Likewise, UCPs, as reactive oxygen species regulators, are involved in the liver responses to toxic injuries by CCl_4_ [23]. In the current results, CCl_4_ injection significantly increased serum AST and ALT level, the expression of CYP2E1 and UCP2 in liver tissue, indicating the induction of liver toxicity.

We found that CCl_4_ injection disrupted liver architecture, hepatocellular edema, centrilobular or periportal necrosis, and destruction of cord-like arrangement of hepatocyte, and acute hepatocyte degeneration. These changes increased the hepatic histopathological score compared to the control group. Eventually, the damage caused by CCl_4_ administration induced liver hypertrophy and increased the liver index. The current findings lead to conclusions similar to previous studies showing liver tissue damage caused by CCl_4_ [2,24].

Liver damage caused by CCl_4_ is characterized by inflammation following generation of reactive oxygen species that cause hepatotoxicity [25]. Induction of excessive reactive oxygen species by CCl_4_ activates Kupffer cells, which mediate liver inflammatory processes through secretion of TNF-α, IL-1β and IL-6 [16,26]. Overproduction of pro-inflammatory cytokines causes inflammation-related liver damage and plays a critical role in the development process [10]. In the current results, increased secretion of TNF-α, IL-1β and IL-6 in serum and liver tissue is due to the damaging effect of CCl_4_. The results of this experiment demonstrate that overproduction of pro-inflammatory cytokines exacerbated the progress of ALI. Since the administration of CCl_4_ profoundly enhanced the secretion of pro-inflammatory cytokines [5], suppression of pro-inflammatory cytokines is an important treatment strategy for acute liver damage.

Stimulation of adenosine A_2A_ receptor in monocytes and macrophages suppresses the production of pro-inflammatory cytokines [18], and agonists of adenosine A_2A_ receptor have been proposed as therapeutic agents for inflammatory diseases [20]. In the current results, PDRN treatment substantially inhibited the secretion of pro-inflammatory cytokines. Inflammation is an adaptive response to cellular or tissue damage, and the MAPK cascade signaling pathway controls the inflammatory response [17]. MAPK is responsible for the regulatory response of pro-inflammatory cytokine production and modulates various inflammatory mediators in most cells [11,13].

Treatment with CCl_4_ activated MAPK to modulate NF-κB [15,25]. NF-κB is rapidly produced in response to reactive oxygen species exposure, stress, and CCl_4_ [16,27]. Degradation of the IκB-α induced by signal initiates the activation of NF-κB, and the activated NF-κB complex generates transcription factors such as TNF-α, IL-1β, and IL-6 [28,29]. In the current results, CCl_4_-induced liver damage enhanced IκB-α phosphorylation, resulting in activation of NF-κB. Activation of NF-κB increased the phosphorylated form of the MAPK cascade, i.e., p-ERK, p-JNK and p-p38 in liver tissue. In contrast, treatment with PDRN reduced IκB-α phosphorylation, resulting in NF-κB inactivation. NF-κB inactivation inhibited the phosphorylated form of the MAPK cascade in liver tissue.

Stimulation of the adenosine A_2A_ receptor enhances intracellular cAMP concentration to inhibit the function of inflammatory neutrophils [30]. The cAMP-PKA pathway is activated by the adenosine A_2A_ receptor to accelerate the phosphorylation of CREB and inhibit inflammatory cytokines through the regulation of NF-κB [31]. Elevated cAMP concentration inhibits the phosphorylated form of the MAPK cascade in stimulated cells [30,32]. In the current results, PDRN treatment enhanced cAMP concentration in CCl_4_-induced ALI mice. The increase in cAMP concentration enhanced phosphorylation of CREP and PKA, suppressed the MAPK cascade, and further inhibited the activation of NF-κB expression in liver tissue. Increased CYP2E1 and UCP2 expression by CCl_4_ administration is associated with inflammation and necrosis following NF-κB/MAPK pathway activation and inhibition of NF-κB/MAPK reduced CYP2E1 and UCP2 expression [22,23,33]. As a result, PDRN treatment also inhibited CYP2E1 and UCP2 expression. For this reason, adenosine A_2A_ receptor agonist PDRN has been suggested as an effective drug for the treatment of inflammatory diseases [19,20]

Excessive apoptosis exacerbates liver damage, and treatment of acute liver disease requires relief of apoptosis [10]. The ratio of Bax vs. Bcl-2 is an important determinant for apoptosis, and DNA fragmentation after exposure to CCl_4_ was observed in liver tissue [22]. The most studied caspases (-3 and -9) are the main factors of apoptosis, and their cleaved forms activate the apoptosis pathway, causing DNA fragmentation to result in cell death [34]. Injection of CCl_4_ has been shown to induce ALI symptoms by increasing apoptosis in liver tissue [5,7]. In the current results, the expression of cleaved caspase-3 and cleaved caspase-9, and Bax vs. Bcl-2 ratio were greatly enhanced after CCl_4_ injection, indicating that CCl_4_ administration accelerated apoptosis. Stimulation of the adenosine A_2A_ receptor enhances cAMP concentration and this enhanced cAMP concentration suppresses apoptotic cell death by releasing anti-apoptosis proteins, which eventually plays a pivotal role in the modulation of cell death [35,36].

In the current study, PDRN treatment inhibited the level of AST and ALT in the serum and liver, the expression of CYP2E1 and UCP2 in liver tissue, and suppressed the liver index and histopathological score. PDRN treatment reduced the expression of cleaved caspase-3 and cleaved caspase-9, and the ratio of Bax vs. Bcl-2 in CCl_4_-induced ALI mice. The enhancing effect of PDRN on cAMP concentration serves as a treatment mechanism for acute liver injury.

As shown in our results, inflammation and apoptosis in the CCl_4_-induced ALI animal model were suppressed by treatment with PDRN. When PDRN was co-treated with the adenosine A_2A_ receptor antagonist DMPX, the anti-inflammatory and anti-apoptotic effects of PDRN disappeared. Therefore, PDRN can be used as an effective treatment for acute liver damage. However, the effectiveness of PDRN on fibrosis, a major cause of liver disease, has not been proven. Anti-fibrotic action is an important factor in the treatment of chronic liver disease. This remains a limitation on the proof of validity of the PDRN. Therefore, it is necessary to evaluate the therapeutic effect of PDRN on chronic liver diseases including cirrhosis in the future.

## 4. Methods

### 4.1. Animals and Classification

Seven-week-old C57BL/6 male mice (*n* = 40) weighing 27 ± 1 g were purchased from a commercial breeder (Orient Bio Co., Seongnam, Korea) and used in this experiment. Using 10 mice per group, the mice were randomly classified into the following four groups: control group, CCl_4_-injection group, CCl_4_-injection and PDRN-treated group, CCl_4_-injection and PDRN with 3,7-dimethyl-1-propargylxanthine (DMPX)-treated group.

This experimental procedure was approved by the Institutional Animal Care and Use Committee of Kyung Hee University and received the following approval number (KHUASP[SE]-17-002). All experimental procedures were performed in good faith in accordance with the guidelines for animal care from the National Institutes of Health and the Korean Institute of Medical Sciences.

### 4.2. Inducing Acute Liver Injury using CCl_4_ and Treatment

**Figure 7:**
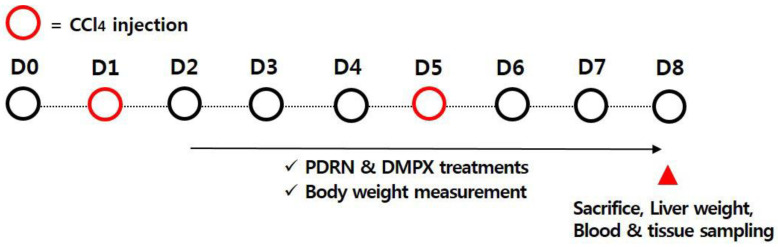
Experimental schedule.

ALI was induced using CCl_4_ in the same manner as described above [37,38]. The mice received an intraperitoneal injection of CCl_4_, and 0.4 mL CCl_4_ dissolved in 100 mL corn oil was injected twice over 7 days at a dose of 10 mL/kg. A single intraperitoneal injection of 200 μL physiological saline containing 8 mg/kg PDRN (Rejuvenex^®^, PharmaResearch Products, Pangyo, Korea) was applied to the mice in the PDRN-treated groups, once a day for seven days, starting one day after the first CCl_4_ injection (Figure 7).

We selected the PDRN concentration that was found to be most effective through previous studies [12,19]. In order to confirm that the action of PDRN occurs through the adenosine A_2A_ receptor, 8 mg/kg DMPX (Sigma-Aldrich Co., St. Louis, MO, USA) was treated with PDRN. For the PDRN-free groups, mice were injected intraperitoneally with 200 µL physiological saline without PDRN on the same schedule.

### 4.3. Blood Sampling, Liver Index, and Tissue Preparation

Blood sampling was performed first, and tissue preparation was conducted in the same manner as described above [12,39]. Mice were weighed before sacrifice and CCl_4_ was first injected and mice were sacrificed 7 days later. After anesthetizing the mice with Zoletil 50^®^ (10 mg/kg; Vibac Laboratories, Carros, France), blood was drawn by cardiac puncture and then maintained at room temperature for 1 h. Blood was centrifuged at 3000 rpm for 20 min to obtain serum.

After blood sampling, physiological saline was perfused through the portal vein, and livers were collected. The wet weight of the mouse liver tissue was measured. The liver index was calculated using body weight and liver wet weight, and the formula is as follows. Liver index = liver weight/body weight ×100 (%). The liver was then fixed in 4% paraformaldehyde, dehydrated with graded ethanol, treated with xylene, infiltrated and embedded in paraffin. Thereafter, coronal sections with a thickness of 5 μm were prepared using a paraffin microtome (Thermo Fisher Scientific, New Jersey, NJ, USA), the sections were mounted on coated slide, and then the slides were dried on a hot plate at 37 °C overnight. An average of 6 slice sections from each liver were collected and used in the next experiment.

### 4.4. Hematoxylin and Eosin (H&E) Staining and Liver Injury Score Analysis

H&E staining for liver histological change was observed in the same manner as described above [19,40]. The slides were soaked in Mayer’s hematoxylin (DAKO, Glostrup, Denmark) for 30 s, washed with water until clean, and again the slides were soaked in eosin (Sigma-Aldrich Co., St. Louis, MO, USA) for 10 s and then washed with water. After air drying, the slides were left at room temperature, then soaked twice each in turn in 95% ethanol, 100% ethanol, 50% ethanol with 50% xylene solution and then 100% xylene. After air drying at room temperature overnight, the slides were mounted on coverslips using Permount^®^ (Thermo Fisher Scientific, New Jersey, NJ, USA).

Histopathological score of liver was evaluated in the same manner as described above [40]. Images from the H&E-stained slides were captured using an Image-Pro^®^ plus computer-assisted image analysis system (Media Cybernetics Inc., Silver Spring, MD, USA) and images were observed with an optical microscope (Olympus, Tokyo, Japan). The images of the slides were evaluated by a blind test of pathologists. The histological score was used to grade the severity of the necrosis and inflammatory process [41,42], as shown in Table 1. The score was calculated by summing all the grades of each item.

### 4.5. Concentration of ALT and AST

ALT and AST concentration was assayed using the ALT and AST activity assay kits (AM 101-K, Asan Pharm Co., Seoul, Korea) from the obtained serum. The Multiskan Go microplate reader (Thermo Fisher Scientific, New Jersey, NJ, USA) was used at absorbance at 510 nm.

### 4.6. Concentration of Pro-Inflammatory Cytokines and cAMP

The concentration of TNF-α, IL-1β, IL-6 (pro-inflammatory cytokines), and cAMP in serum and liver tissue was detected by ELISA using the enzyme immunoassay kit (Abcam, Cambridge, UK) in the same manner as described above [19,43].

### 4.7. Western Blot Analysis

NF-κB activation and phosphorylation of signaling factors of MAPK were measured by Western blot in the same manner as described above [19]. Moreover, the expression of cleaved caspase-3-positive cells and cleaved caspase-9-positive cells was analyzed by the same method. Liver tissue was homogenized in cooled RIPA buffer (Cell Signaling Technology, Beverly, MA, USA) using 1 mM PMSF (Sigma-Aldrich Co., St. Louis, MO, USA), and next the homogenate was centrifuged at 14,000 rpm for 30 min at 4 °C. Protein amount was determined using a μ-drop reader (Thermo Fisher Scientific, New Jersey, NJ, USA), and 30 μg protein was separated on an SDS-PAGE gel and transferred to a nitrocellulose membrane. The following reagents were used as the primary antibodies: anti-rabbit antibody for NF-κB (1:1000; Abcam, Cambridge, UK), for IκB-α (1:1000; Santa Cruz Biotechnology, Santa Cruz, CA, USA), for phosphorylated (p)-IκB-α (1:1000; Santa Cruz Biotechnology, Santa Cruz, CA, USA), for ERK (1:2000; Cell Signaling Technology, Beverly, MA, USA), for p-ERK (1:2000; Cell Signaling Technology, Beverly, MA, USA), for JNK (1:2000; Cell Signaling Technology, Beverly, MA, USA), for p-JNK (1:2000; Cell Signaling Technology, Beverly, MA, USA), for p38 (1:2000; Cell Signaling Technology, Beverly, MA, USA), for p-p38 (1:2000; Cell Signaling Technology, Beverly, MA, USA), for cleaved caspase-3-positive cells (1:2000; Cell Signaling Technology, Beverly, MA, USA), for cleaved caspase-9-positive cells (1:2000; Cell Signaling Technology, Beverly, MA, USA), for CYP2E1 (1:2000; Enzo Life Sciences, Inc., New York, NY, USA), and anti-mouse antibody for UCP2 (1:1000; Santa Cruz Biotechnology, Santa Cruz, CA, USA), for Bax (1:1000; Santa Cruz Biotechnology, Santa Cruz, CA, USA), for Bcl-2 (1:1000; Santa Cruz Biotechnology, Santa Cruz, CA, USA), for β-actin (1:1000; Santa Cruz Biotechnology, Santa Cruz, CA, USA). The membrane was treated with the secondary antibody, horseradish peroxidase (HRP)-conjugated IgG (1:2000; Vector Laboratories, Burlingame, CA, USA), and bands were detected by an enhanced chemiluminescence (ECL) detection kit (Bio-Rad, Hercules, CA, USA). In order to compare the protein expression, the detected bands were computed densitically by Image-Pro^®^ Plus computer-assisted image analysis system (Media Cybernetics Inc., Silver Spring, MD, USA). The control group was set at 1.00 for relative quantification.

### 4.8. Data Analysis

Multiple comparisons were performed using SPSS software (version 23.0, IBM Co., Armonk, NY, USA). Statistical analysis among the groups was conducted using one-way ANOVA followed by Tukey’s HSD post-test. The data were presented as the mean ± standard error of the mean, and *p* < 0.05 showed statistically significance.

## Figures and Tables

**Table 1:** Histological scoring parameters.

Grade	Periportal Bridging Necrosis	Centrilobular Degeneration and Necrosis	Portal Inflammation
0	None	None	None portal inflammation
1	Mild piecemeal necrosis	Mild (ballooning degeneration and/or scattered foci of hepatocellular necrosis in <20% of lobules or nodules)	Mild (sprinkling of inflammatory cells in <20% portal tracts)
2	Moderate piece necrosis (involves less than 50% of the circumference of most portal tracts)	Moderate (involvement of 20~40% of lobules or nodules)	Moderate (increased inflammatory cells in 20~40% of portal tracts)
3	Marked piecemeal necrosis (involves more than 50% of the circumference of most portal tracts)	Marked (involvement of 50% of lobules or nodules)	Marked (increased inflammatory cells in 50% of portal tracts)
4	Marked piecemeal necrosis plus bridging necrosis	Very marked (involvement of >60% of lobules or nodules)	Very marked (dense packing of inflammatory cells in >60% of portal tracts

## Abbreviations

ALI	Acute liver injury
ALT	Alanine aminotransferase
AST	Aspartate aminotransferase
Bax	Bcl-2-associated X protein
Bcl-2	B-cell lymphoma-2
cAMP	cyclic adenosine-3′,5′-monophosphate
CCl_4_	Carbon tetrachloride
CREB	cAMP response element-binding protein
CYP2E1	cytochrome P450 2E1
DMPX	7-dimethyl-1-propargylxanthine
ELISA	Enzyme-linked immunoassay
ERK	Extracellular signal-regulated kinase
H&E	Hematoxylin and eosin
IL-1β	Interleukin-1β
IL-6	Interleukin-6
IκB-α	Nuclear factor-κB inhibitor-α
JNK	c-Jun N-terminal kinase
MAPK	Mitogen-activated protein kinases
NF-κB	Nuclear factor-κB
PDRN	Polydeoxyribonucleotide
PKA	Protein kinases A
TNF-α	Tumor necrosis factor-α
UCP2	uncoupling protein2
